# Development, implementation, and feasibility of site-specific hepatitis C virus treatment workflows for treating vulnerable, high-risk populations: protocol of the Erase Hep C study — a prospective single-arm intervention trial

**DOI:** 10.1186/s40814-023-01311-4

**Published:** 2023-05-08

**Authors:** Anmol Desai, Lauren O’Neal, Kia Reinis, Patrick Chang, Cristal Brown, Michael Stefanowicz, Audrey Kuang, Deepak Agrawal, Darlene Bhavnani, Tim Mercer

**Affiliations:** 1 Department of Population Health, The University of Texas at Austin Dell Medical School, Austin, USA; 2 The University of Texas at Austin Dell Medical School, Austin, USA; 3 Department of Internal Medicine, The University of Texas at Austin Dell Medical School, Austin, USA; 4 CommUnityCare Health Centers, Austin, USA

**Keywords:** Hepatitis C virus, Homelessness, Intravenous drug use, Implementation science, Simplified treatment, Site-specific treatment, Direct-acting antivirals, Sustained virological response, Healthcare for the Homeless, People who inject drugs

## Abstract

**Background:**

Hepatitis C virus (HCV) is the leading indication for liver transplantation and liver-related mortality. The development of direct-acting antivirals (DAA) and a simplified treatment algorithm with a > 97% cure rate should make global elimination of HCV an achievable goal. Yet, vulnerable populations with high rates of HCV still have limited access to treatment. By designing locally contextualized site-specific HCV treatment workflows, we aim to cure HCV in vulnerable, high-risk populations, including people experiencing homelessness (PEH) and people who inject drugs (PWID), in Austin, TX, USA.

**Methods:**

Our implementation science study will utilize a qualitative and design thinking approach to characterize patient and systemic barriers and facilitators to HCV treatment in vulnerable, high-risk populations seeking care across seven diverse primary care clinics serving PEHs and PWIDs. Qualitative interviews guided by the Practical, Robust Implementation and Sustainability Model (PRISM) framework will identify barriers and facilitators by leveraging knowledge and experience from both clinic staff and patients. Data synthesized using thematic analysis and design thinking will feed into workshops with clinic stakeholders for idea generation to design site-specific HCV treatment workflows. Providers will be trained on the use of a simplified HCV treatment algorithm with DAAs and clinic staff on the new site-specific HCV treatment workflows. These workflows will be implemented by the seven diverse primary care clinics serving vulnerable, high-risk populations. Implementation and clinical outcomes will be measured using data collected through interviews with staff as well as through medical chart review.

**Discussion:**

Our study provides a model of how to contextualize and implement site-specific HCV treatment workflows targeting vulnerable, high-risk populations in other geographic locations. This model can be adopted for future implementation research programs aiming to develop and implement site-specific treatment workflows for vulnerable, high-risk populations and in primary care clinical settings for other disease states beyond just HCV.

**Trial registration:**

Registered on *ClinicalTrials.gov* on July, 14, 2022. Identifier: NCT05460130.

**Supplementary Information:**

The online version contains supplementary material available at 10.1186/s40814-023-01311-4.

## Background

Hepatitis C virus (HCV), the leading indication of liver-related mortality, can be effectively cured with oral direct-acting antivirals (DAAs), making global elimination of HCV by 2030 an achievable goal [1]. However, only a minority of the total population infected with HCV has access to care or has received treatment [2]. Elimination of HCV will require expanding access to treatment in vulnerable, high-risk populations, such as people experiencing homelessness (PEH) and people who inject drugs (PWID). Systematic reviews have demonstrated that HCV prevalence is 32% among PEH in the United States of America (USA) and 55% among PWID in North America [3]. PEH and PWID are populations that may overlap; the prevalence of HCV among PEH seeking care at Health Care for the Homeless (HCH) clinics in the USA is estimated to be 31% and 70% among those who also inject drugs [4].

HCV treatment in primary care settings is as effective as treatment traditionally provided by specialists, which has been demonstrated in the general population and among those with opioid use disorder receiving opioid agonist therapy [5, 6]. Among PEH, HCV treatment in primary care settings is both feasible and cost-effective [7–10]. Nurse-led HCV treatment models demonstrate that PEH can successfully be retained in care through treatment to cure [11]. Use of a novel, simplified HCV treatment algorithm by primary care physicians and mid-level practitioners (nurse practitioners and physician assistants) can increase capacity and be used to scale-up HCV treatment [1]. Evidence-based principles of this simplified treatment algorithm include eliminating sobriety requirements, using pan-genotypic treatments, minimizing unnecessary lab monitoring, and facilitating medication access via street teams or care navigation approaches [1, 12–16].

The Erase Hep C study takes this new, simplified treatment algorithm one step further through both the provision of treatment in diverse primary care settings and by targeting primary care clinics serving vulnerable, high-risk populations, including PEH and PWID. An implementation science approach, consistent with a hybrid type 1 study design, will be used to develop locally contextualized site-specific HCV treatment workflows and evaluate the feasibility and effectiveness of these workflows in achieving cure in these vulnerable, high-risk populations [17].

### Aims and objectives

The purpose of the Erase Hep C study is to implement and evaluate the implementation of site-specific HCV treatment workflows by newly trained frontline healthcare providers across multiple diverse primary care clinics serving vulnerable, high-risk populations in Austin, TX, USA. The primary aim is to cure chronic HCV infection in patients seeking care at these primary care clinics in Austin, TX, USA.

To achieve this primary aim, the following four objectives will be pursued (Table 1).

**Table 1:** Erase Hep C study objectives

Objective	Approach
1. Identify facilitators and barriers affecting HCV treatment workflows	Conduct a contextual assessment that includes observational process mapping and qualitative interviews with patients, clinic staff, and external organizations that provide HCV testing and either linkage to care or provide treatment in the Austin area
2. Design site-specific HCV treatment workflows using baseline data from objective 1	A human-centered design approach will be used to co-create these workflows in a series of collaborative site-specific workshops between the research and clinic-specific teams
3. Develop and deliver a curriculum to train frontline primary healthcare providers on the evidence-based, simplified HCV treatment algorithm and to train providers and clinic staff on the site-specific HCV treatment workflows	Utilize the simplified HCV clinical curriculum developed by the research team’s hepatologist and the workflows designed in objective 2
4. Conduct a prospective single-arm clinical trial of these site-specific HCV treatment workflows to evaluate both clinical and implementation outcomes	Implement the workflows designed in objective 2. Enroll and treat 289 patients to achieve cure of HCV

## Methods/design

### Setting

The study will be implemented at seven primary care clinics within CommUnityCare (CUC), Austin’s largest federally qualified health center (FQHC) network, which houses Austin’s Health Care for the Homeless program. The study sites were selected because of their focus on care for vulnerable, high-risk populations for HCV, including PEH and PWID. The study site locations include the following: (1) a full-spectrum primary care clinic located within the Austin Resource Center for the Homeless (ARCH) shelter (“the ARCH Clinic”); (2) a patient-centered medical home providing full-spectrum primary care for PEH as well as other medically complex and socially vulnerable patients following hospital discharge (“care connections”); (3) CUC’s dedicated clinic providing buprenorphine to individuals with opioid use disorder (medication-assisted therapy (MAT) clinic — “the MAT Clinic”); (4) a street medicine team brings care to PEH, meeting them at homeless campsites and under bridges (“the street team”); (5) a clinic located at Community First! Village (CFV), a permanent supportive housing community for individuals who were chronically homeless (“the CFV Clinic”); (6) a clinic located at Esperanza Community, a state-sanctioned encampment for PEH that is becoming a transformational shelter complex (“the Esperanza Clinic”); and (7) a full-spectrum primary care clinic space setup within Sunrise Church, providing care alongside the local mental health authority and other social service providers that partner with the Sunrise Homeless Navigation Center nonprofit that serves PEH in Austin (“Sunrise”).

### Implementation framework

**Fig. 1:**
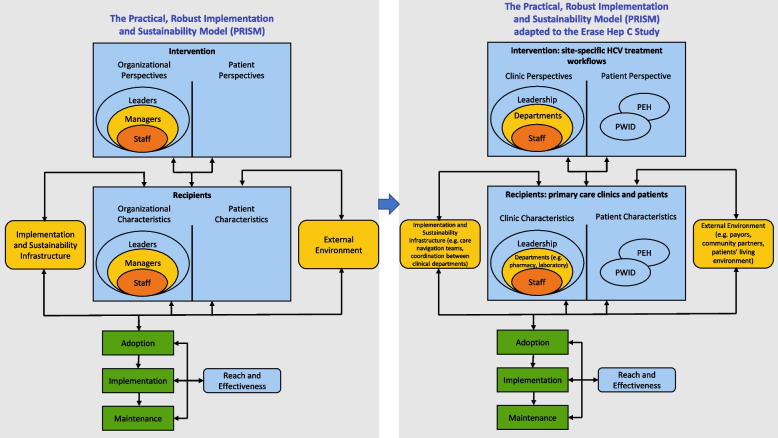
PRISM Implementation Science Framework and adaptation to the Erase Hep C study

The Practical, Robust, Implementation and Sustainability Model (PRISM) will guide the study implementation. PRISM is an evidence-based implementation science framework which integrates the perspective of organizational managers, frontline staff, patients, and key external stakeholders and recognizes the importance to translate research into operations (Fig. 1) [18]. We have adapted the PRISM framework to the Erase Hep C study to show how patient, organizational, infrastructure, and environmental characteristics affect the design and implementation of site-specific HCV treatment workflows in primary care clinics. The PRISM framework will be applied to the design of the observational process mapping and semi-structured interview questions and will also help guide the qualitative analysis, allowing us to identify contextual issues that could affect implementation of our site-specific HCV treatment workflows. The PRISM outcome measures are guided by the RE-AIM framework, a robust, well-tested framework for measuring both individual patient-level and organizational-level outcomes to evaluate the implementation of the site-specific HCV treatment workflows across five key dimensions: reach, effectiveness, adoption, implementation, and maintenance [19].

#### Objective 1: Baseline contextual assessment

First, we will conduct a baseline assessment to identify contextual facilitators and barriers affecting implementation of HCV treatment at each of our clinical sites. We will use a design thinking and qualitative approach consisting of observational process mapping and qualitative key informant interviews. We will also create an HCV care cascade to outline current treatment workflows throughout the time course of caring for a patient with HCV, from diagnosis to cure.

#### Baseline observational process mapping

We will engage in observational process mapping, guided by the PRISM framework, to become familiar with the clinic culture and build relationships and trust with clinic staff, in preparation to being in clinic regularly during the enrollment period. We will receive provider-guided tours of each clinical site to understand the clinic space, staffing structures and ratios, and observe the clinic setting and culture, including clinic staff behaviors, interactions with each other, patients, and external community partners, as well as understand where within the community the primary care clinic is located. We will also observe providers and clinic staff in their roles, through their day-to-day activities, how they make decisions, and their communication styles, to learn clinic organization processes and map each clinic’s treatment and operational workflow.

#### Baseline qualitative interviews

Guided by the PRISM framework, we will develop and conduct semi-structured qualitative interviews with clinic staff and patients to leverage individual experience and knowledge to identify barriers and facilitators of HCV care. The interview guides will include questions addressing all constructs of the PRISM domains: organization characteristics, characteristics of the organization’s staff, patient characteristics, implementation and sustainability infrastructure, and external environment. Key informant interviews will be held to elucidate a broad set of perspectives and generate consensus on how the current HCV treatment workflow can be improved or adapted, given the patient population, organizational characteristics, and external environment related to each clinical site.

The qualitative interviews will be conducted across the study sites with a purposive sample of 10–15 patients diagnosed with HCV falling anywhere along the HCV care cascade, from treatment naïve to cured; 25–30 CUC staff (physicians, nurse practitioners, nurses, pharmacists, medical assistants, care coordinators/case managers, and health system administrators/leaders); and staff from approximately five organizations external to CUC that test and either link to care or provide HCV treatment. The research assistant or research coordinator will conduct the key informant interviews. Each interview will last approximately 30 to 60 min and will be audio-recorded for verbatim transcription.

#### Baseline data analysis

Field notes will be taken during the observational process mapping, and guided by the PRISM framework, a set of a priori codes will be developed. The qualitative interviews will be double coded and analyzed using thematic descriptive and interpretive coding techniques. Building on the a priori codes developed from the observational process mapping, a structured codebook with definitions and examples organized by the PRISM framework will be iteratively developed from themes that emerge from the interviews.

Two coders will independently conduct line-by-line analysis of interview transcripts, comparing similarities and differences in behaviors, activities, settings, experiences, emotions, and meanings that are expressed across interviews. The coders will then come together to discuss, negotiate, and revise the codes. This will be an iterative process to collaboratively develop a final codebook that outlines the definition and application of each code. Following codebook development, the coders will apply the finalized codebook to all interviews. All the interview data will be coded using the qualitative data management and analysis software, NVivo version 12.

The results will be synthesized using a human-centered design thinking approach. Results will be presented as a product that allows for iterative collaboration with clinic staff and the translation of findings to site-specific HCV treatment workflows, as described below.

#### Objective 2: Design workshops and site-specific HCV treatment workflow development

Using baseline data from the observational process mapping and qualitative interviews, we will engage key stakeholders in a participatory, human-centered design process to develop site-specific HCV treatment workflows for each of the seven clinical sites. Leveraging knowledge from The University of Texas at Austin’s Design Institute for Health in an advisory capacity, and building off previous experience using human-centered design in implementation research, our research team will facilitate two 1-h design workshops with each clinic-specific team and other CUC staff members involved in supporting HCV care [20]. The design process will include reflection, validation, prioritization, brainstorming, conceptualization, and creation of the site-specific HCV treatment workflows.

In the first design workshop, we will discuss the synthesized interview findings so clinic-specific teams can validate our generalized thematic results across all clinical sites and any site-specific factors from the baseline assessment. Then, each clinic-specific team will prioritize powerful facilitators and critical barriers to HCV care and brainstorm actionable solutions for HCV treatment at their clinical sites.

In the second design workshop, the clinic-specific teams will evaluate the advantages and disadvantages of ideas generated from brainstorming and create a more concrete HCV treatment workflow with actionable solutions specific to each site. These site-specific HCV treatment workflows will be built on the scaffolding of the evidence-based, simplified HCV treatment algorithm and overarching principles recently published from proceedings of a consensus meeting of leading, international HCV experts, along with expert input from a local hepatologist and other local CUC HCV treatment providers [1]. The potential principles of the simplified HCV treatment algorithm include treatment that is as follows: decentralized to the primary care setting with flexible scheduling, eliminates sobriety or advanced cirrhosis requirements for drug approval, reduces unnecessary lab testing, uses pan-genotypic regimens to approach a test and treat model, facilitates drug delivery and/or treatment via street medicine or community-based care models to overcome access barriers, provides HCV treatment alongside opioid agonist therapy, and provides care coordination, patient tracking, and data capture to maximize retention and minimize those lost to follow-up.

Starting with the backbone of these common elements, driven by the evidence-based, simplified HCV treatment algorithm, each site will make adaptations based on their clinic’s staffing ratio, physical space, team dynamics, and patient population characteristics. Each sites’ adaptations will be captured and layered on top of the backbone of common elements to create the site-specific HCV treatment protocols.

#### Objective 3: Provider training

Frontline healthcare providers and nurses from each of our clinical sites, some who have never treated HCV and others who have treated but not using the low-barrier-simplified treatment algorithm, will be trained on the evidence-based, simplified HCV treatment algorithm and the locally contextualized site-specific HCV treatment workflows. The hepatologist co-investigator on the research team will develop the curriculum for the evidence-based simplified treatment algorithm and deliver the training. Training will be conducted in a 1-h session, accompanied by printed and open-source online materials for the trained providers to take home and use at point of care. The curriculum will cover HCV pathophysiology, screening, diagnosis, laboratory and radiographic evaluation, treatment regimens, treatment effectiveness data from clinical trials, side effects, and drug interactions. Specific training of our study’s inclusion and exclusion criteria, and the site-specific HCV treatment workflows, will be delivered with concept reinforcement through case-based learning. Training material will also cover difficult cases and special populations, with additional emphasis on determining if hepatologist consultation is needed, and when patients should be referred to a specialist. The hepatologist co-investigator also provides care within the CUC clinical network and, along with other specialized HCV providers at CUC, is available for ongoing clinical consultation to primary care providers as needed.

#### Objective 4: Prospective single-arm intervention trial

By designing and adapting HCV treatment workflows for each specific clinical site and training frontline healthcare providers, we hypothesize that we can effectively treat HCV across multiple primary care clinics serving vulnerable, high-risk populations. We plan to enroll 289 patients over 6 months. Following the baseline assessment, design of the site-specific HCV treatment workflows, and training of frontline healthcare providers, we will implement the workflows across our seven clinical sites and measure both clinical and implementation outcomes. The SPIRIT figure shows the timeline of enrollment, intervention, and assessments for the trial (Table 2). The SPIRIT checklist is provided in Additional file 1.

**Table 2:** Erase Hep C study SPIRIT figure

	Screen	Enrollment	Treatment				
			Start	End (Glecaprevir / Pibrentasvir)	End (Sofosbuvir / Velpatasvir)	SVR12 (Glecaprevir / Pibrentasvir)	SVR12 (Sofosburvir / Velpatasvir)
Timepoint	***T***_**-1**_	***T***_**0**_	***T***_**1**_** = up to 90 days after *T***_**0**_	***T***_**2**_** = *T***_**1**_** + 60 days**	***T***_**3**_** = *T***_**1**_** + 90 days**	***T***_**4**_** = *T***_**2**_** + (90–120 days)**	***T***_**5**_** = *T***_**3**_** + (90–120 days)**
ENROLLMENT:							
Eligibility Screen	X						
Informed Consent		X					
Interview		X		X	X		
Obtain EMR Data	X	X	X	X	X	X	X
INTERVENTION:							
Implement Site-Specific HCV Treatment Workflows		X	X	X	X	X	X
ASSESSMENTS:							
Demographic Variables		X		X	X		
Socioeconomic Variables		X		X	X		
Substance Use Variables		X		X	X		
Sexual Behavior Variables				X	X		
Medical History		X		X	X		
Labs	X	X		X	X		
SVR12						X	X
2º Clinical Outcomes			X	X	X	X	X
2º Implementation Outcomes			X	X	X	X	X

### Study participants

Adult patients who are 18 years or older have been laboratory diagnosed with chronic hepatitis C (HCV antibody positive with detectable viral load) and are enrolled in care at one of CUC’s seven clinical sites participating in the study which will be eligible for inclusion in the trial. Exclusion criteria will be applied when identifying patients eligible for enrollment. Exclusion criteria include the following: clinically decompensated cirrhosis (history or current presence of ascites, hepatic encephalopathy or variceal hemorrhage, or Child Pugh score ≥ 7); non-treatment naïve, including previous treatment with either DAAs or interferon-based regimens; status-post liver transplant or actively on the transplant list awaiting transplant; human immunodeficiency virus (HIV) or hepatitis B virus (HBV) co-infection; currently pregnant; inability to provide informed consent; and provider or patient desire to be referred to a specialist. These criteria will be used in order to exclude complex cases from study participation. Complex cases will be referred to specialists and may still be offered treatment, as per standard of care.

#### Identifying eligible patients

The majority of patients recruited into the study will be existing patients at our clinical sites with known HCV infection, but who have not yet accessed HCV treatment. Other patients eligible for the study will be either new patients to the clinical sites or existing patients who have not been previously screened and are newly diagnosed with HCV.

A report will be generated from CUC’s electronic medical record (EMR) system to identify patients with chronic HCV infection. The research team will then conduct a chart review of each patient who may be eligible for inclusion in the study, discussing with the clinical teams, as needed. Additionally, the research team’s hepatologist will conduct a clinical review to determine if patients with cirrhosis are compensated and meet inclusion criteria. Frontline healthcare providers and nurses who are trained on the inclusion and exclusion criteria may assist in identifying eligible patients and refer them to research team members rotating between the clinical sites to enroll study participants. Additionally, the research team will note when patients have upcoming clinic appointments and will plan to be available to meet patients at the clinic before or after their visit.

#### Standard of care

Patients will be screened and tested for HCV as part of the standard of care. Patients will be screened prior to their identification by the research team. Patients are screened using an antibody test that, if positive, will reflex to a quantitative RNA PCR test. Patients positive on both tests are diagnosed with chronic HCV infection.

Treatment for HCV will be offered as part of the clinical standard of care. Recruitment into the study will not depend on whether the study participant is offered or receives treatment. Taking a population-based approach, we will enroll all eligible patients who have been diagnosed with chronic HCV infection, not just those who are offered and accept treatment.

#### Intervention

Our study intervention will be the implementation of site-specific HCV treatment workflows. Ad hoc consultation with a hepatologist is already available within the healthcare system, and providers can continue to reach out as needed. This will help ensure high-quality care while reducing unnecessary and expensive specialist visits that further perpetuate barriers to accessing HCV treatment for vulnerable, high-risk patient populations.

### Data collection

Data collection will occur using two main methods: (1) interviews with study participants and (2) data extraction from CUC’s EMR. Data will be extracted from the EMR at enrollment, upon treatment completion, and 12 weeks after treatment completion, when viral load is measured to determine cure, known as sustained virologic response at 12 weeks, or SVR12. Variables collected from the EMR will include the following: HCV RNA PCR test results; primary care physician; laboratory test results used to determine eligibility, including those for HIV (HIV antibody)and HBV (hepatitis B surface antigen); demographic information such as age, sex, gender, race, and ethnicity; medical comorbidities excluding psychiatric diagnoses (the latter will be collected directly from patients); prescriptions for HCV treatment; orders for an HCV genotype test and ultrasound (as “unnecessary” tests to measure fidelity); and dates of medication pickup. Date of first medication pickup will be the proxy for date of treatment initiation.

Data will be captured and stored in REDCap, a HIPAA-compliant, secure data collection tool and database that only the research team members access. Participants’ identifiable data will be retained to enable contact through to the end of the study. Identifiers and identifiable data will be retained for future research on HCV if the study participant provides written informed consent to do so. Since this is a minimal risk study, a data safety monitoring board is not required. The research team will utilize an internal data dashboard to monitor the study progress and data quality. Quality of data collection will also be tracked by the research team. Data quality measures will consist of (1) weekly checks of missing and incomplete data collected from interviews and the EMR by the research assistant and research coordinator and (2) automated monthly reports of the frequency of missing and incomplete data. In some instances, if we find that data from the EMR is frequently missing, the research team may need to discuss collecting the data directly from the patient.

#### Enrollment

Study participants will be enrolled over a 6-month period. Study participants will interact with the research team at one or two time points: upon enrollment and, among those who go on treatment, at the end of treatment completion, which may be approximately 2–3 months from enrollment.

At the time of the patient’s clinic visit, a member of the research team will meet with the patient to describe the study and obtain written informed consent from those willing to participate. Clinic staff will provide an introduction of the eligible patient to the research team, as needed. Research team members will also recruit pre-screened patients from around the clinic setting with the help of clinic staff. The team will work with clinic staff to position themselves within the clinics so as to minimize disruption of workflows and clinical care.

On a biweekly basis, the research team will examine study progress by reviewing enrollment at each study site, including basic demographics of study participants, to ensure enrollment is occurring as intended. The research assistant and research coordinator will monitor study progress weekly. Additionally, we will track reasons why study participants were not treated using the site-specific HCV treatment workflows (e.g., changes in clinical status, lost to follow-up from the clinic). We will also generate estimates of how many study participants are at each step along the HCV care cascade. There will be specific thresholds to trigger an ad hoc review if enrollment is not occurring as intended.

#### First interview at enrollment

The first interview will be conducted by a research team member at time of study enrollment. The face-to-face interview, lasting approximately 20 min including enrollment time, will include questions on medical coverage, education, social support, work, housing, substance use history, mental health disorders and psychiatric diagnoses, and contact information.

#### Second interview at treatment completion

Regimens typically require 8 or 12 weeks of treatment. Upon treatment completion, a laboratory test is performed to measure HCV viral load. This test is repeated 12 weeks after treatment completion (SVR12) to ensure that the response is sustained and the patient is cured of HCV. A viral load < 15 IU/mL is considered undetectable. Record of a laboratory test taken at the time of treatment completion will serve as a proxy for treatment completion. The second, shorter interview will ideally occur when the participants have their blood drawn for this test at treatment completion. Follow-up data will be collected on substance use and sexual behavior during treatment, medication adherence, and treatment side effects. If needed, research team members may reach out to the study participant by phone to arrange a time to meet for the second interview.

In the absence of test results, other markers for treatment completion may be used. These include self-report, date of last bottle pickup + 28 days, or clinical documentation of treatment completion in a note in the EMR. Study participants who do not pick up all treatment doses or do not return for a final laboratory test for viral load will be considered lost to follow-up and will be assumed to have not achieved SVR12.

### Evaluation of study clinical and implementation outcomes

#### Clinical outcomes

The primary outcome will measure the proportion of patients with chronic HCV infection enrolled in the study that achieves SVR12 (cure) as defined by an undetectable viral load < 15 IU/mL at 12 weeks or later after treatment completion.

The secondary clinical outcomes measure is as follows:

1. Time (in days) from being offered treatment to initiating treatment, among those enrolled in the study
2. Proportion of patients enrolled in the study who initiate HCV treatment
3. Proportion of patients enrolled in the study who complete HCV treatment

#### Implementation outcomes

The research team will measure key implementation outcomes to evaluate how the new site-specific HCV treatment workflows are utilized, integrated, and maintained in real-world organizational structures across our clinical sites. Key implementation outcomes include reach, effectiveness, adoption, implementation, and maintenance, as defined by the evidence-based RE-AIM framework [18]. Analyses of these outcomes will inform the potential for “real-world” scale-up of the HCV treatment intervention in the routine practice of other primary care clinical settings and across healthcare systems.

Strategies for measuring RE-AIM outcomes are shown in Table 3. Our primary and secondary clinical outcomes will measure effectiveness. We will use a combination of data collected from qualitative interviews and quantitative data from chart review to evaluate the implementation outcomes. Reach and effectiveness will be measured quantitatively using data extracted from the EMR and our REDCap database of patient-reported outcomes. Adoption will be measured quantitatively by extracting this data from REDCap. Additionally, we will conduct follow-up qualitative interviews with one or two clinical staff at each site to get a qualitative assessment of the implementation, maintenance, and acceptability of the adapted site-specific HCV treatment workflows to the clinic-specific teams. A convenience sample of 1–2 staff (provider and MA or nurse) from each site (7–14 total sample) will be used. Interviews will be audio-recorded and transcribed for analysis. For implementation, we will develop a rubric to measure fidelity to the site-specific HCV treatment workflows and will perform a fidelity assessment by chart review on a random sample of 10% of patients enrolled in the trial. The EMR will be reviewed for each patient to see how closely these workflows were implemented as intended, including examining the appropriateness, timing, and frequency of labs ordered.

**Table 3:** RE-AIM measures

Implementation outcome	Measurement strategy	Data collection methodology
Reach	Reach of the site-specific HCV treatment workflows: the proportion of study participants who are offered HCV treatment *(Offered HCV treatment will be measured by the presence of a prescription for an HCV medication in the EMR)*	Primary data collection EMR chart review
Effectiveness	Primary outcome: the proportion of study participants that achieve SVR12 Secondary outcomes: the proportion of study participants that complete treatment. The proportion of study participants that initiate treatment	Primary data collection
Adoption	The proportion of providers who are trained per site	Primary data collection
Implementation	The extent to which the site-specific HCV treatment workflows are implemented as intended (fidelity) for study participants Markers include the following: the proportion of study participants who were genotyped, among study participants who reached SVR12. The proportion of study participants for whom an ultrasound was ordered, among non-cirrhotic study participants who reached SVR12	EMR chart review and qualitative interviews with clinic staff
Maintenance	The extent to which the site-specific HCV treatment workflows are sustained over the study’s 16-month data collection period	EMR chart review and qualitative interviews with clinic staff

### Sample size justification

We predict that we will achieve SVR12 in 75% of patients enrolled in our study. We will use the “intention-to-treat” principle by including those enrolled in the study and lost to follow-up in the denominator. Therefore, the denominator will consist of the population of patients at participating clinical sites who have been screened and diagnosed with chronic HCV infection and who have provided informed consent to participate in our study, regardless of whether treatment was initiated or not. These estimates were based on experience treating HCV in this patient population, as well as three published studies of HCV treatment interventions for people experiencing homelessness in the primary care setting [9, 10, 21].

**Fig. 2:**
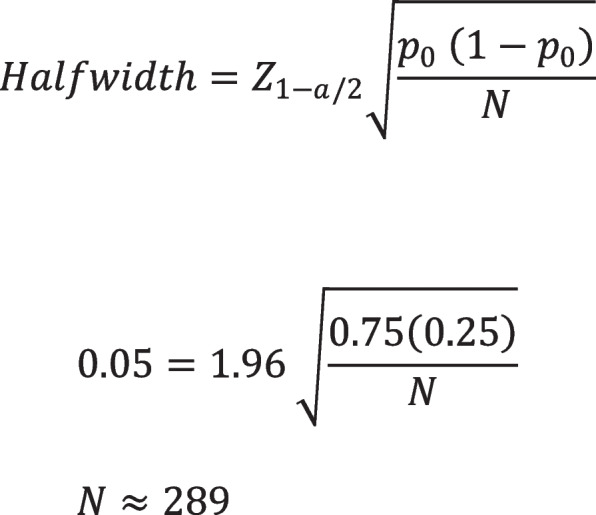
Sample size calculation

With the expectation that 75% of patients enrolled in the study will achieve SVR12, a two-sided 95% confidence interval will be constructed with a half-width no more than 5% from the point estimate. In order to assure with 95% confidence that the true proportion achieving SVR12 under the locally contextualized site-specific HCV treatment workflows is within 5% of the observed proportion, the required sample size is 289 patients (Fig. 2).

### Analytical approach

We will quantify the proportion of the study population with HCV that achieved SVR12, including a confidence interval for this proportion, using the “intention-to-treat” principle as detailed above. Secondary analyses will follow the “per-protocol” approach where the denominator will exclude participants lost to follow-up.

Participants who do not pick up all their HCV medication and who do not complete a final laboratory test for viral load by the end of the trial period will be recorded as not having completed treatment, considered lost to follow-up, and assumed to have not achieved SVR12. Participants who either miss or delay treatment doses or who do not pick up all treatment doses, but return for a treatment completion lab or SVR12, will be retained within the dataset and recorded as non-adherent for descriptive analyses. We will also evaluate the number and proportion of patients moving through each step of the HCV care cascade, from diagnosis of HCV and enrollment to achieving SVR12 in order to better understand barriers within the care cascade. General reasons for failure to initiate and complete treatment will be discussed with clinic staff and summarized, as will delays between each step of the care cascade.

To derive proportions and 95% confidence intervals (95% CI), we will apply generalized estimating equations (GEE) to logistic regression models without predictors and interpret the anti-logit of the intercept as our proportion. Bias-corrected GEE will be applied to account for a small sample size. We will examine characteristics of the patient population stratified by achievement of SVR12. These characteristics will include age, sex, race, ethnicity, housing factors, education, employment status, payor, mental health conditions and psychiatric diagnoses, substance use history, sexual behavior, medical comorbidities, clinical site, and adherence to HCV treatment. These characteristics will also be evaluated after adjusting for covariates using a multivariable logistic regression model. Reported outcomes will include adjusted odds ratios for SVR12 and their 95% confidence intervals. We will use GEE models to account for clustered data and correct for small sample size, as described above. We will also conduct similar exploratory analyses for our secondary outcomes.

Implementation outcomes will be analyzed descriptively. For the qualitative portion of the RE-AIM implementation outcomes, we will double code and analyze the audio-recorded and transcribed interviews using thematic descriptive and interpretive coding techniques. Secondary analysis will be done for some of the RE-AIM implementation outcomes. Secondary analysis for effectiveness may include percent of study participants who reached SVR12 that were non-adherent (missed or delayed doses). Secondary analysis for adoption may include two measures: (1) proportion of study participants initiating treatment of all offered, where the proxy for offered treatment is submitting a written prescription, and (2) proportion of study participants who pick up the first bottle of medication as a proxy for initiating treatment, therefore adopting the site-specific HCV treatment workflows. Secondary analysis for implementation will examine how fidelity affects the primary outcome in the aggregate.

## Discussion

HCV has become increasingly easier to cure, yet many vulnerable populations with high rates of HCV still remain untreated. Elimination of HCV will require targeted expansion of treatment in vulnerable, high-risk populations, specifically PEH and PWID.

The Erase Hep C study expands upon the evidence-based, simplified HCV treatment algorithm by targeting primary care clinics serving vulnerable, high-risk populations, including PEH and PWID, in Austin, TX, USA. By designing locally contextualized site-specific HCV treatment workflows, we aim to make treatment and cure easier for these patients and for the clinical teams providing their care. We will evaluate the feasibility and effectiveness of these workflows in achieving cure in these populations. As an implementation science study, we do not have a plan for promoting participant retention in the study that would go beyond what the primary care clinics can continue after the study is complete. However, working with a hepatologist already on CUC staff will empower frontline healthcare providers to provide effective HCV treatment in the primary care setting, enhancing the sustainability of our HCV treatment model after the study period ends.

The Erase Hep C study has the potential to inform the expansion of HCV treatment to other vulnerable, high-risk populations and clinical settings. Our study protocol describes an implementation research approach utilizing a design-thinking and qualitative baseline assessment to adapt site-specific HCV treatment workflows to make treatment easier and more accessible for vulnerable, high-risk populations. Through a human-centered design thinking process informed by our baseline assessment, we aim to show how site-specific HCV treatment workflows can be implemented into the existing organizational workflows and infrastructure of primary care clinics caring specifically for PEH and PWID, in order to expand access to HCV treatment. By using a population health approach to define our denominator as patients eligible for treatment and consented to participate in our study, we broaden our reach compared to other studies which only included patients who initiated treatment [9, 10, 21].

The participatory engagement method we use in the baseline contextual assessment and design workshops to bring in clinic level staff engagement will speak to success of the feasibility of the study. Measuring implementation outcomes, specifically adoption and maintenance, will help assess the feasibility of scaling up implementation of these site-specific, simplified HCV treatment workflows within this healthcare system and how it could work in other healthcare systems.

Our study does have some limitations. First, there may be resistance from clinic staff to participate in the baseline assessment and the implementation of the site-specific HCV treatment workflows. The research coordinator and research assistant will spend time at each clinic to build relationships and garner buy-in from clinic staff. The co-primary investigators and co-investigators are also clinicians within CUC’s clinical network and at several of our study sites, and they will further foster buy-in and participation from clinical staff. Additionally, we will learn about the existing treatment and operational workflows to better integrate the site-specific HCV treatment workflows in the existing clinic workflows, reducing any additional work for clinical staff. Lastly, by nature of the human-centered design workshops, the site-specific HCV treatment workflows are co-created between the research team and clinic staff, generating shared ownership of the research process.

Second, by nature, this patient population is hard to reach and often do not return to the clinic, which may be a limitation to recruitment and enrollment. Building strong relationships with the clinic staff will help build trust with the patients as well. We also address this limitation by starting our site-specific HCV treatment workflow process at diagnosis, once the patient is already in the clinic. Analytically, we will capture this in our intent-to-treat approach generating a real-world picture of engagement along the HCV care cascade.

Third, this is a single-arm trial and lacks a control group. This was a trade-off between research design and clinical integration, and our analytic approach and measurement of implementation outcomes provide additional strength to this real-world study. However, future randomized controlled implementation trials may be needed to further generalize our results.

Fourth, we recognize that this healthcare system has specialists embedded within it, and providers have access to consultation with hepatologists or specialized primary care providers, which may not be accessible within all healthcare systems. There may be limitations to scalability for healthcare systems that do not have access to hepatologists. However, there are tools available for these healthcare systems, such as the free Gastrointestinal & Hepatobiliary Consultation Service through the University of California San Francisco or utilizing a local Hepatitis C Project ECHO (Extension for Community Healthcare Outcomes) [22, 23].

Despite these limitations, through evaluation of the clinical and implementation outcomes, we aim to develop an approach that could serve as a model for future implementation research programs aiming to develop and implement site-specific HCV treatment workflows for vulnerable, high-risk populations in primary care clinics. Additionally, we expect our study to demonstrate that contextualizing the evidence-based, simplified HCV treatment algorithm will facilitate better access for vulnerable, high-risk populations to cure HCV. This approach could be used for other disease states beyond just HCV.

### Trial status

The baseline assessment, human-centered design workshops, and provider training have been completed. Enrollment into the trail started in September 2022. This is study protocol version 2.3, and the version date is November 18, 2022.

## Supplementary Information

**Additional file 1.** SPIRIT 2013 Checklist: Development and implementation of site-specific Hepatitis C Virus treatment workflows for treating vulnerable, high-risk populations: protocol of the Erase Hep C study, a prospective single-arm intervention trial.

## Data Availability

Upon completion of our trial, we will make de-identifiable data available to researchers and other stakeholders working to expand access to HCV treatment upon request. Identifiable data of those who provided written informed consent for future use may be shared with collaborating researchers in future studies. Data collection instruments and statistical code can be made available upon request.

## Abbreviations

HCV: Hepatitis C virus
DAA: Direct-acting antivirals
PEH: People experiencing homelessness
PWID: People who inject drugs
HCH: Health Care for the Homeless
CUC: CommUnityCare
FQHC: Federally Qualified Health Center
ARCH: Austin Resource Center for the Homeless
MAT: Medication-assisted therapy
CFV: Community First! Village
PRISM: Practical, Robust, Implementation and Sustainability Model
HIV: Human immunodeficiency virus
HBV: Hepatitis B virus
EMR: Electronic medical record
SVR12: Serologic Viral Load 12 weeks after treatment completion
WHO: World Health Organization
IRB: Institutional review board
NIH: National Institutes for Health
ECHO: Extension for Community Healthcare Outcomes
