# Correction: Development, implementation, and feasibility of site‑specific hepatitis C virus treatment workflows for treating vulnerable, high‑risk populations: protocol of the Erase Hep C study — a prospective single‑arm intervention trial

**DOI:** 10.1186/s40814-023-01347-6

**Published:** 2023-06-30

**Authors:** Anmol Desai, Lauren O’Neal, Kia Reinis, Patrick Chang, Cristal Brown, Michael Stefanowicz, Audrey Kuang, Deepak Agrawal, Darlene Bhavnani, Tim Mercer

**Affiliations:** 1 Department of Population Health, The University of Texas at Austin Dell Medical School, Austin, USA; 2 The University of Texas at Austin Dell Medical School, Austin, USA; 3 Department of Internal Medicine, The University of Texas at Austin Dell Medical School, Austin, USA; 4 CommUnityCare Health Centers, Austin, USA

**Correction: Pilot Feasibility Stud 9, 78 (2023)**

**https://doi.org/10.1186/s40814-023-01311-4**

Following publication of the original article [1], the authors identified an error in Table 2. The correct table is given below.

**Table 2:** Erase Hep C study SPIRIT figure

	Screen	Enrollment	Treatment				
			Start	End (Glecaprevir /Pibrentasvir)	End (Sofosbuvir /Velpatasvir)	SVR12 (Glecaprevir /Pibrentasvir)	SVR12 (Sofosbuvir /Velpatasvir)
TIMEPOINT	**T**_**-1**_	**T**_**0**_	**T**_**1**_** = up to 90 days after T**_**0**_	**T**_**2**_** = T**_**1**_** + 60 days**	**T**_**3**_** = T**_**1**_** + 90 days**	**T**_**4**_** = T**_**2**_** + (90–120 days)**	**T**_**5**_** = T**_**3**_** + (90–120 days)**
ENROLLMENT:							
Eligibility Screen	X						
Informed Consent		X					
Interview		X		X	X		
Obtain EMR Data	X	X	X	X	X	X	X
INTERVENTION:							
Implement Site-Specific HCV Treatment Workflows		X	X	X	X	X	X
ASSESSMENTS:							
Demographic Variables		X		X	X		
Socioeconomic Variables		X		X	X		
Substance Use Variables		X		X	X		
Sexual Behavior Variables				X	X		
Medical History		X		X	X		
Labs	X	X		X	X		
SVR12						X	X
2º Clinical Outcomes			X	X	X	X	X
2º Implementation Outcomes			X	X	X	X	X

The original article [1] has been updated.

## References

[1] Desai A, O’Neal L, Reinis K (2023). Development, implementation, and feasibility of site-specific hepatitis C virus treatment workflows for treating vulnerable, high-risk populations: protocol of the Erase Hep C study — a prospective single-arm intervention trial. Pilot Feasibility Stud.

